# 0.04 degree-per-hour MEMS disk resonator gyroscope with high-quality factor (510 k) and long decaying time constant (74.9 s)

**DOI:** 10.1038/s41378-018-0035-0

**Published:** 2018-11-19

**Authors:** Qingsong Li, Dingbang Xiao, Xin Zhou, Yi Xu, Ming Zhuo, Zhanqiang Hou, Kaixuan He, Yongmeng Zhang, Xuezhong Wu

**Affiliations:** 1 College of Intelligence Science, National University of Defense Technology, Changsha, 410073 China; 2 Laboratory of Science and Technology on Integrated Logistics Support, National University of Defense Technology, Changsha, 410073 China; 3 State Key Laboratory of ASIC and System, School of Microelectronics, Fudan University, Shanghai, 200433 China; 4 East China Institute of Photo-Electron IC, Bengbu, 233042 China

## Abstract

The disk resonator gyroscope is an attractive candidate for high-performance MEMS gyroscopes. This gyroscope consists of a sensor and readout electronics, and the characteristics of the sensor directly determine the performance. For the sensor, a high-quality factor and long decaying time constant are the most important characteristics required to achieve high performance. We report a disk resonator gyroscope with a measured quality factor of 510 k and decaying time constant of 74.9 s, which is a record for MEMS silicon disk resonator gyroscopes, to the best of our knowledge. To improve the quality factor of the DRG, the quality factor improvement mechanism is first analyzed, and based on this mechanism two stiffness-mass decoupled methods, i.e., spoke length distribution optimization and lumped mass configuration design, are proposed and demonstrated. A disk resonator gyroscope prototype is fabricated based on these design strategies, and the sensor itself shows an angle random walk as low as 0.001°/√h, demonstrating true potential to achieve navigation-grade performance. The gyroscope with readout electronics shows an angle random walk of 0.01°/√h and a bias instability of 0.04°/h at room temperature without compensation, revealing that the performance of the gyroscope is severely limited by the readout electronics, which should be further improved. We expect that the quality factor improvement methods can be used in the design of other MEMS gyroscopes and that the newly designed DRG can be further improved to achieve navigation-grade performances for high-end industrial, transportation, aerospace, and automotive applications.

## MEMS: high-quality factor sensors for resonator gyroscopes

A disk resonator gyroscope demonstrates a high-quality factor of 510 k and decay time constant of 74.9 s. High-performance MEMS gyroscopes are in demand for a range of high-end applications. Disk resonator gyroscopes are particularly promising candidates due to inherent mode matching and high thermal stability. However, many applications demand high-quality factors and long decay times. Now, a team led by Xuezhong Wu at the National University of Defense Technology, China, reports what they claim are record quality factors and decay times for MEMS silicon disk gyroscopes. Two mechanisms for stiffness-mass decoupling are attributed to the high performance, which the authors say could be used for the design of other MEMS gyroscopes.

## Introduction

High-performance microelectromechanical system (MEMS) gyroscopes are in wide demand in high-end industrial, transportation, aerospace, and automotive applications. Considerable research has been conducted on MEMS Coriolis vibratory gyroscopes (CVGs), such as disk^1–13^, bulk acoustic wave^14^, quad mass^15^, and microscale 3D wineglass gyroscopes^16,17^. Among these, the disk resonator gyroscope (DRG) is an attractive candidate for high-performance MEMS gyroscopes due to its inherent mode matching, high thermal stability, low anchor loss, and abundant electrodes. The first 0.01°/h navigation-grade MEMS DRG with active temperature control and compensation was previously reported^1^ and demonstrated the potential of DRGs for use in high-performance MEMS gyroscopes.

A high-quality factor (Q), long decaying time constant (*τ*), high symmetry of damping, and mode matching are the essential attributes of high-performance CVGs^1,16,17^. In MEMS CVGs, a quad mass gyroscope (QMG) with a *Q* value >1.7 million and *τ* longer than 328 s, has been reported^15^. A bulk acoustic wave (BAW) gyroscope with *Q* up to 1.38 million has been measured at a 2.745 MHz center frequency^14^. A *Q* factor as high as 4.45 million and a decaying time constant longer than 259 s have been demonstrated on a fused silica birdbath shell resonator^17^. For DRGs, the state-of-art of the reported highest *Q* was 0.358 million, and the longest decaying time constant was 38.5 s in our previous report^18^; these values severely limit the potential performance improvement. Therefore, it is necessary to further improve the *Q* and *τ* of DRGs, for which *τ* = *Q*/*πf*_0_ and *f*_0_ is the resonant frequency.

The quality factor is a measure of the energy dissipation in vibrating resonators. Several energy dissipation mechanisms exist, including air damping, surface loss, support loss, electrical damping, and thermoelastic damping (TED)^19–23^. In DRGs encapsulated in high-vacuum packages and working in elliptical modes, TED has been identified as the major energy dissipation mechanism and imposes an upper limit on the attainable *Q*^8,24^. Therefore, thermoelastic quality factor (*Q*_TED_) improvement is the main target in optimization of DRGs. The TED can be separated into two categories: material-dependent and geometry-dependent components^25–27^. For MEMS DRGs, which are always manufactured with silicon, the material-dependent component has been fixed, and thus high *Q*_TED_ mechanical design becomes a highly significant issue. Certain examples of *Q*_TED_ improvement exist via innovative structural design or structural optimization for DRGs. Reference studies^8,12^ have presented a method for *Q*_TED_ improvement by lengthening the spokes in DRGs and manipulating the heat flux paths in the resonator. Our previous work proposed a method for improving the *Q*_TED_ of DRGs by optimizing the ring thickness distribution^28–30^.

However, in most research, the *Q*_TED_ improvement mechanisms were not clearly explained, and the *Q* and *τ* of DRGs can only be slightly improved. In this paper, we present the *Q*_TED_ improvement mechanism in the DRG, and according to this mechanism, two novel stiffness-decoupled methods are proposed for *Q* and *τ* improvement. The first method is stiffness distribution optimization achieved by spoke length distribution (SLD) optimization, in which the spoke lengths on different layers of the DRG are designed to be varied, instead of identical in the traditional DRGs, and the optimum SLD is obtained using the particle swarm optimization (PSO) method. The other method is mass distribution optimization achieved by the lumped mass configuration, in which the lumped masses are hung on the ring frame to increase the effective mass while maintaining the stability of the effective stiffness. The effectiveness of the two methods is demonstrated with the finite element method (FEM) and the experimental results. The newly designed DRG based on these design strategies is fabricated and characterized. The *Q* and *τ* are tested as 510 k and 74.9 s, respectively, which are the records for MEMS DRGs. The theoretical angle random walk (ARW) of the DRG sensor itself is as low as 0.001°/√h, showing a potential to achieve navigation-grade performance. In contrast, the ARW and bias instability of the gyroscope are tested as 0.01°/√h and 0.04°/h, respectively, showing that the noise and instability of the gyroscope are severely dominated by the readout electronics. Improvement of the readout electronics could further reduce the ARW and instability of the gyroscope for high-end applications. Furthermore, the *Q* and *τ* improvement methods proposed in this paper can be broadly used in the optimization of MEMS-vibrating gyroscopes and other resonators.

## Results

### Model and operation of the DRG

**Fig. 1:**
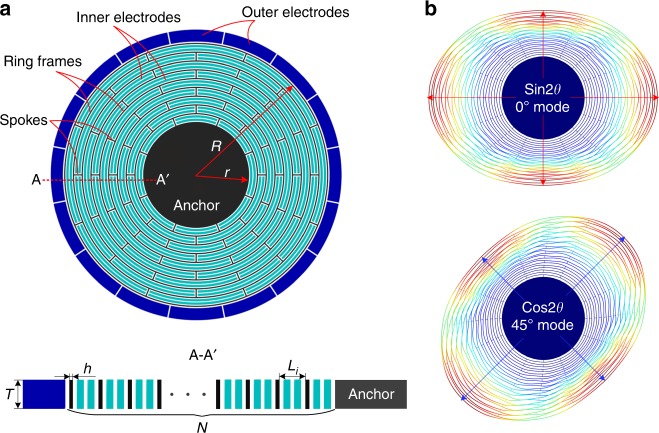
**a** Schematic view of the traditional disk resonator gyroscope. **b** Working modes of the DRG

**Fig. 2:**
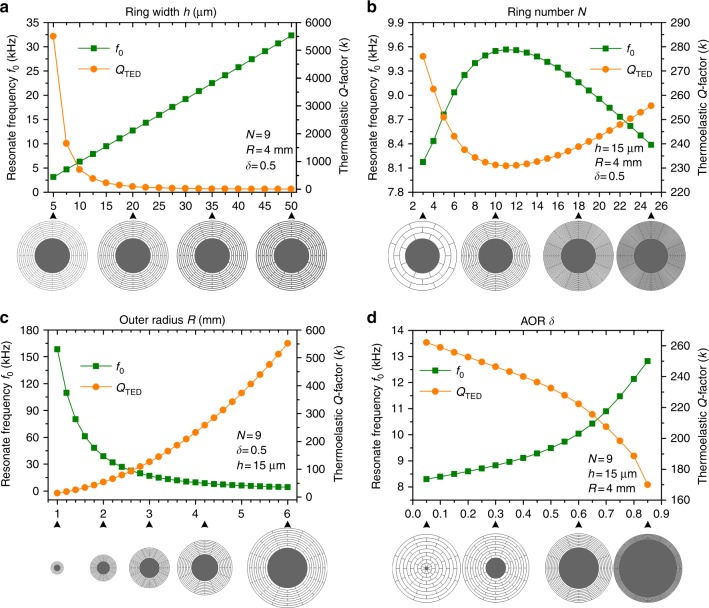
**a** Effects of geometrical parameters on the resonant frequency and *Q*_TED_ of the DRGs. Ring width varying from 5 μm to 50 μm, **b** ring numbers varying from 3 to 25, **c** outer radius varying from 1 mm to 6 mm, and **d** outer radius to anchor radius ratio varying from 0.05 to 0.85

The traditional DRG shown in Fig. 1a consists of a disk resonator, 16 outer electrodes, and layers of inner electrodes. The disk resonator is composed of several concentric ring frames interconnected through eight spokes and a single central anchor. The device thickness, ring number, outer radius, anchor radius, anchor radius to outer radius ratio (AOR), and ring width are defined as *T*, *N*, *R*, *r*, *δ*(*r/R*), and *h*, respectively. The disk resonator is excited at the *n* = 2 elliptical driving mode, and the Coriolis force is detected at the degenerate sensing mode, which is located geometrically 45° apart from the driving mode, as shown in Fig. 1b. Ideally, the two modes have an identical natural frequency but indistinguishable mode shapes.,

Due to the complexity of the relationships between the geometrical parameters and the DRG characteristics, the effects of the geometrical parameters on the DRG *Q*_TED_ and *f*_0_ are investigated using FEM. The results are shown in Fig. 2. It can be observed that the ring width and outer radius are the main factors affecting the *Q*_TED_ in the DRG, whereas the ring number and AOR have slight influences. Therefore, for the traditional DRGs with identical ring width and ring gaps, to improve the *Q*_TED_, the ring width should be as small as possible and the outer radius should be as large as possible. However, the ring width is limited by the fabrication capability, and the outer radius is limited by the device size. Thus for the traditional DRGs, *Q*_TED_ improvement will come to an end only by changing the basic geometrical parameters *h*, *N*, *R*, and *δ*. In this paper, two novel methods are presented to break the limit.

### *Q*_TED_ improvement mechanism in DRGs

**Fig. 3:**
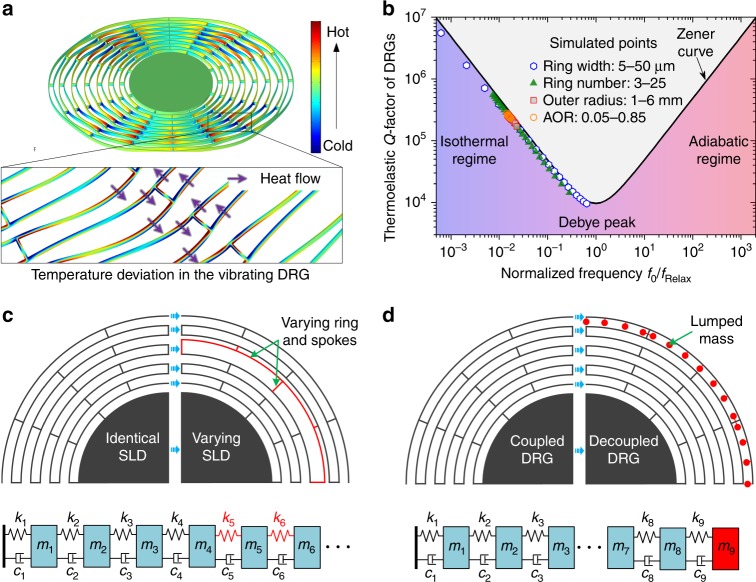
**a** Temperature deviation of the full device and heat flow in the beams at the working modes. **b** Zener curve and comparison of the TED in DRGs with different geometrical parameters obtained by the Zener model and COMSOL simulation. **c** Schematic of the stiffness distribution optimization method: spoke length distribution optimization. **d** Schematic of the mass distribution optimization method: lumped mass configuration

The temperature deviation and heat flow in the vibrating DRG are shown in Fig. 3a, illustrating that the main thermoelastic dissipation originates from the in-plane bending deformation of the ring frames and spokes in the DRG. The disk resonator can be viewed as the combination of thin beams with different length and shapes. Thus, it can be assumed that the *f*–*Q* relationship of DRGs can be expressed with the widely applied Zener one-dimensional TED theoretical model:^25,26^

1 $$Q_{{\mathrm {TED}}} = \frac{{2C_{\mathrm v}}}{{E\alpha ^2T_0}} \cdot \frac{{1 + \left( {f_0/f_{{\mathrm {Relax}}}} \right)^2}}{{2\left( {f_0/f_{{\mathrm {Relax}}}} \right)}}$$

2 $$f_{ {\mathrm {Relax}}} = \frac{{\pi k}}{{2h^2C_{\mathrm v}}}$$

Herein, *f*_0_ and *f*_Relax_ are resonant frequency and thermal relaxation frequency, respectively; *E* is the Young’s modulus; *α* is the linear coefficient of thermal expansion; *T*_0_ is the equilibrium temperature of the beam; *C*_v_ is the specific heat per unit volume; *k* is the thermal conductivity of the beam; and *h* is the beam width in the flexural direction. The Zener curve and the simulated results for the different geometrical parameters presented in Fig. 2 are compared in Fig. 3b, showing that the simulated *Q* values are slightly lower than the theoretical *Q* due to neglect of the out-of-plane energy dissipation. However, in the design of DRGs, slight errors can be neglected compared with the other errors. Thus the Zener 1D TED model can be used in analysis of the TED in DRGs.

The Zener curve shows that the maximum dissipation occurs at the Debye peak, where *f*_0_ = *f*_Relax_. In the low-frequency range *f*_0_≪*f*_Relax_, the strain rate is reduced and the temperature gradient is consequently reduced, leading to low levels of energy dissipation, and thus the system is considered isothermal. In the high-frequency range *f*_0_≫*f*_Relax_, notably low levels of energy are dissipated, similar to the low-frequency range, and the system is adiabatic. Because most of the DRGs operate in the low-frequency range, it can be observed that the key to improving *Q*_TED_ in DRGs is to minimize the normalized frequency *f*_0_/*f*_Relax_. For the DRG with a certain ring width, *f*_Relax_ is fixed, and thus the most effective way to improve *Q*_TED_ is to decrease its resonant frequency *f*_0_.

The DRG with *N* rings can be treated as the combination of *N* resonators coupled in a series of different stiffness and masses, as depicted in Fig. 3c, d where *m*_*i*_ is the mass of the rings and *k*_*i*_ is the effective stiffness affected by the ring width, ring radius, and spoke lengths. Improvement of *Q* by changing the basic geometrical parameters reaches a limit because the stiffness and mass of the DRG are coupled with each other. This paper proposes two novel methods to decouple the stiffness and mass of the DRG and further improve the *Q* factor of DRGs. One method is SLD optimization, in which the spoke lengths are changed to primarily affect the stiffness distribution of the DRG and further change the resonant frequency, as depicted in Fig. 3c. The other method is lumped mass configuration, in which the lumped masses are hung on the ring frame to primarily affect the mass distribution of the DRG and also change the resonant frequency, as depicted in Fig. 3d. This paper aims to find the optimum SLD and the lumped mass configuration to further improve the quality factor of the DRG.

### SLD optimization

To simplify the analysis, a nine-ring DRG model is used. The length of the spokes on the *i*th layer of the ring is defined as *L*_*i*_. This optimization is applied under the condition that the other structural parameters *R*, *r*, *h*, and *N* are fixed. Thus, the total sum of the spoke lengths should be constant:

3 $$\mathop {\sum}\limits_{i = 1}^n {L_i = R - r - Nh} = Sum$$

Additionally, the spoke lengths should be limited in a range from *a* to *b*:

4 $$\begin{array}{*{20}{c}} {a \le L_i \le b} & {(i = 1,2,3...n)} \end{array}$$

Herein, the ring number *N* is 9, and *R*, *r*, *h*, *a*, and *b* are set as 4 mm, 1.9 mm, 12 μm, 0.2 mm, and 0.6 mm, respectively, according to the actual requirement of the device size and the fabrication capability.

Stochastic evolutionary algorithms are proposed and widely applied to solve multidimensional design optimization problems. For example, the genetic algorithm (GA) was used in multidisciplinary system design of microgyroscopes^31^, ant colony optimization (ACO) was applied for structural topology optimization^32^, and PSO was chosen for structural optimization of MEMS resonators for *Q* factor improvement^33^. In this paper, the PSO method^34,35^ is used to find the optimum SLD for the highest *Q*_TED_ because of its simple formula and powerful function. The SLD can be expressed as *L* = (*L*_1_, *L*_2_, *L*_3_, *L*_4_, *L*_5_, *L*_6_, *L*_7_, *L*_8_, and *L*_9_). Each distribution can be viewed as a nine-dimensional location of a particle, and *Q*_TED_ is an implicit function of *L* that can be treated as the fitness value in PSO. A total of ten particles and 50 iterations are selected as a trade-off between computing time and tolerance. The steps in the PSO process are listed as follows: (a) randomly initialize the positions and velocities of all particles in the problem space shown in Equations (4) and (5); (b) calculate the fitness value (*Q*_TED_) of each particle using COMSOL Multiphysics; (c) for every particle, compare the current fitness value with the *pbest* (the best fitness this particle has achieved so far) and update *pbest* with the largest value; (d) for every particle, compare the current fitness with the *gbest* (the best fitness ever searched by all of the particles in the previous iterations) and update *gbest* with the largest value; (e) update the velocity of the particle according to the following:

5 $$\begin{array}{ccccc}\\ v_j^{k + 1} = \omega v_j^k + c_1{\mathrm {Rand}}(0,1)(pbest - x_j^k)\\ \\ \,\,\,\,\,\,\,\,\,\,\, + c_2{\mathrm {Rand}}{\prime}(0,1)(gbest - x_j^k)\\ \end{array}$$

where *v*_*j*_^*k*^ and *x*_*j*_^*k*^ are the velocity and position of the *j*th particle at the *k*th iteration, Rand(0,1) and Randʹ(0,1) are two separately generated random numbers within [0,1], *ω* is the inertia weight, *c*_1_ and *c*_2_ are acceleration coefficients, and in this investigation, we set *c*_1_, *c*_2_, and *ω* to 1.49, 1.49, and 0.729, respectively; (f) update the position of the particle according to the following

6 $$x_j^{k + 1} = x_j^k + v_j^{k + 1}$$

(g) Loop to step (b). The iteration is terminated when sufficiently good fitness or the maximum number of iterations is achieved.

**Fig. 4:**
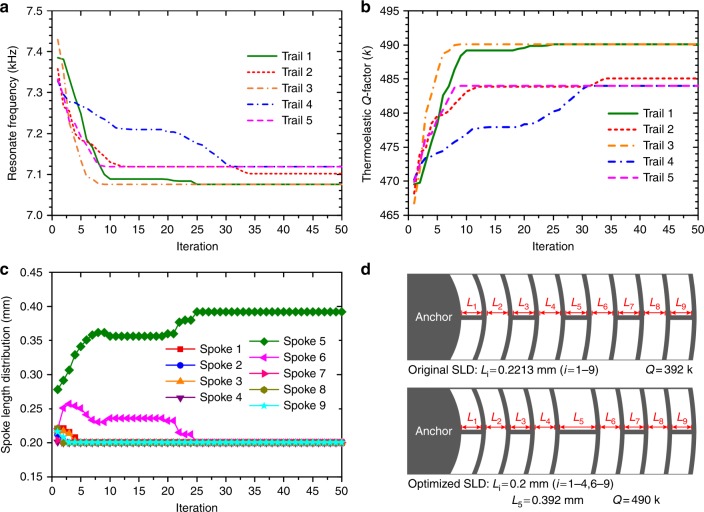
**a, b** Plots of the resonant frequency *f*_0_ and *Q*_TED_ of the DRG at each iteration in the PSO progress. **c** Plot of the spoke length distributions at each iteration in trail 1. **d** Schematic view of the spoke length distribution in the traditional nine-ring DRG and the DRG with optimized SLD

The PSO processes of the natural frequency *f*_0_ and *Q*_TED_ of the nine-ring DRG are shown in Fig. 4a, b. Five trails are shown, and trails 1 and 3 converge to the largest optimum. The SLD optimization processes in trail 1 are shown in Fig. 4c, and the optimized SLD is shown in Fig. 4d. It can be observed that the global optimum is achieved when the lengths of spokes 1–4 and 6–9 reach the lower boundary of 0.2 mm, whereas the length of spoke 5 is the residual of the constant *Sum* and the other spoke lengths, 0.392 mm. Additionally, the global optimum *Q*_TED_ is ∼490 k, which is a 25% improvement compared with the *Q*_TED_ of the traditional DRG with the identical spoke length (392 k).

### Lumped mass configuration

**Fig. 5:**
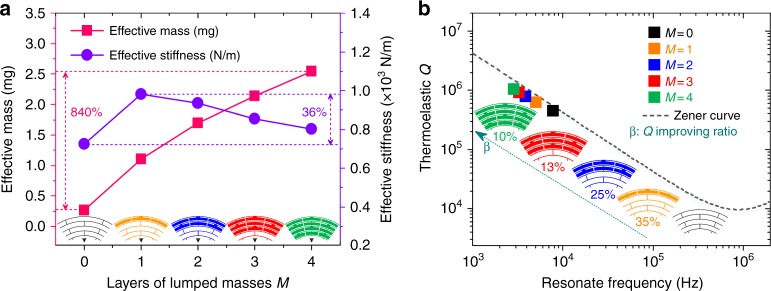
**a** Effects of the layers of the lumped masses on the effective mass and stiffness of the DRG. **b** Effects of the layers of the lumped masses on the resonant frequency and *Q*_TED_ of the DRG

In the lumped mass configuration, the masses are hung on the ring frames and spokes with a single beam. The lumped masses can greatly change the masses in the DRGs while slightly affecting the effective stiffness. As shown in Fig. 5a, with the increase of the lumped masses layers from 0 to 4, the effective mass shows an 8.4-fold improvement, whereas the effective stiffness only changes by up to 36%. Thus the resonant frequency can be greatly decreased, while the thermal relaxation frequency remains nearly constant. The thermoelastic dissipation in the lumped masses can be neglected because their elastic deformation is notably small. Thus, the Zener 1D TED model is still suitable for the stiffness-mass decoupled DRG, as shown in Fig. 5b. From Fig. 5b, it can also be observed that with increase of the lumped mass layers, *Q*_TED_ is improved. However, the improvement ratio decreases gradually from 35% to 10% because the lumped masses hanging on the outer rings have a larger contribution to the effective mass improvement than the lumped masses hanging on the inner rings. The layers of lumped masses are set to four as a trade-off between the *Q* factor and the number of inner electrodes. The inner electrodes can be used to improve the capabilities of driving, sensing, and tuning of the DRG.

### Model and prototype of the new DRG

**Fig. 6:**
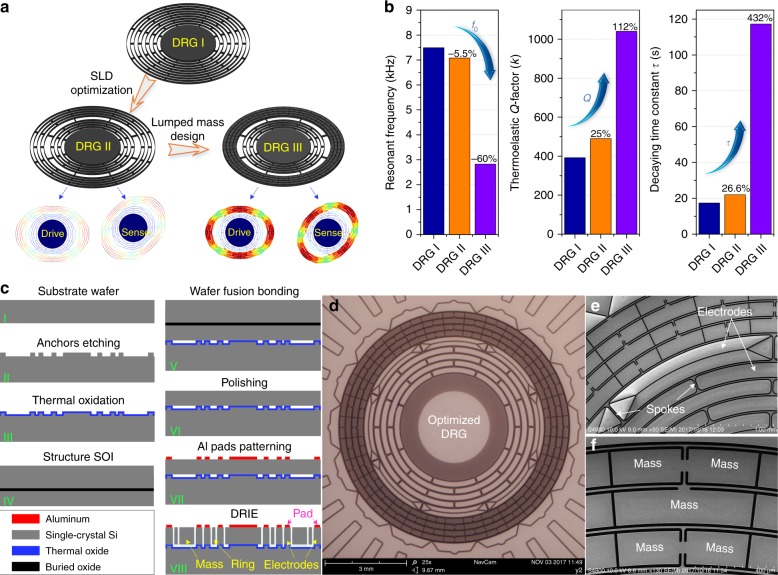
**a** Schematic of the resonators in the traditional DRG, DRG with the optimized SLD, and DRG with both optimized SLD and lumped mass configuration. **b** Comparison of the resonant frequency, *Q*_TED_ and decaying time constant for the three types of DRGs. **c** Fabrication process of the optimized DRG. **d** Photograph of the fabricated optimized DRG. **e** SEM view of the spokes and inner electrodes of the optimized DRG. **f** SEM view of the lumped masses

The schematics of the resonators in the traditional DRG (DRG I), the DRG with SLD optimization (DRG II), and the final design using both optimization methods (DRG III) are shown in Fig. 6a. The resonant frequency *Q*_TED_ and the decaying time constant τ of the three types of DRGs are compared in Fig. 6b. This figure shows that with use of the SLD optimization, *Q* and *τ* of the DRG are improved by ∼25% and ∼26.6%, respectively. Furthermore, using the stiffness-mass decoupled design, *Q* and *τ* of the DRG are greatly improved by 112% and 432%, respectively. These improvements can directly reduce the bias instability and mechanical noise of the gyroscope. This disk resonator is fabricated using wafer fusion bonding and deep reactive ion etching (DRIE) processes. Detailed introductions to the fabrication processes are supplied in the Materials and Methods section. The schematics of the fabrication processes and images of the fabricated DRG are shown in Fig. 6c–f.

### Gyroscope performance characterization

**Fig. 7:**
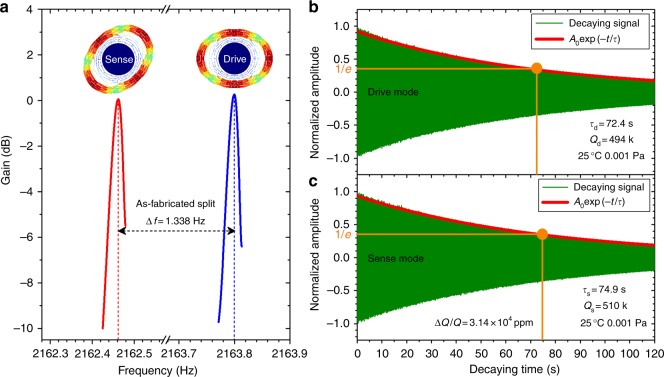
**a** Frequency response of the driving and sensing modes in the optimized DRG. **b**, **c** Ring-down testing results of the drive mode and sense mode for the DRG

To characterize the properties of the optimized sensor, the frequency response and ring-down tests are implemented. Using the frequency response analyzer (NF FRA5087), the frequency responses of the driving and sensing modes of the DRG are obtained as shown in Fig. 7a, showing that the initial frequency split between the two modes is approximately 1.338 Hz (Δ*f*/*f*_0_ = 600 ppm). For the high *Q* resonator, the frequency-domain *Q* measurement is often difficult and inaccurate due to nonlinear effects. Therefore, the ring-down technique is used in this work for *Q* factor measurement. The ring-down testing results are shown in Fig. 7b, c. The figure shows that *Q* of the optimized DRG reaches 510 k and the decaying time constant reaches 74.9 s. To the best of our knowledge, this result presents the highest *Q* and longest decaying time constant demonstrated in MEMS silicon DRGs to date. This result also demonstrates that the optimization methods proposed in this paper are effective for *Q* and *τ* improvement in DRGs. A discrepancy between the measured and simulated quality factors reveals that the other damping factors in DRGs can no longer be neglected if the *Q* is greater than 500 k. Because the air pressure is sufficiently low, air damping can be neglected. Thus, residual damping mainly originates from the support damping, surface damping, and electrical damping, which should be reduced in future research.

**Fig. 8:**
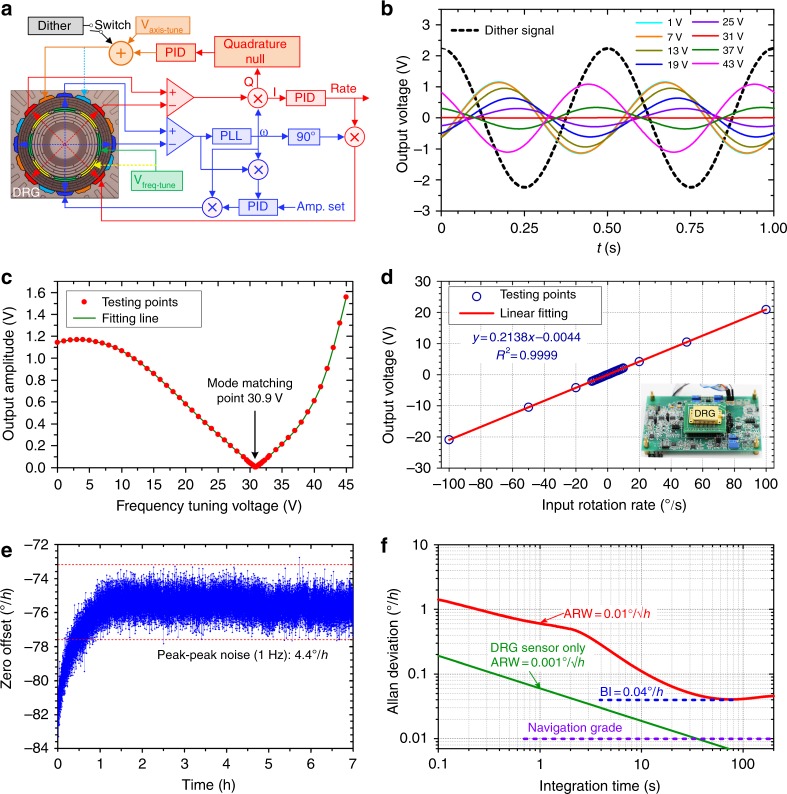
**a** Readout circuit of the optimized DRG. **b** Gyroscope output signal and **c** output amplitude due to the quadrature electrode dither at various frequency-tuning voltages ranging from 0 to 45 V. **d** Output response of the DRG to rotation rate range from −100°/s to 100°/s. **e** Bias offset test of the DRG for 7 h at room temperature. **f** Allan deviation plot of the optimized DRG and the sensor only

The schematic of the readout circuit is shown in Fig. 8a. The gyroscope is driven to fixed displacement with the phase-locked loop (PLL) and proportional integral derivative (PID) controller. The drive/sense axes misalignment is tuned with the quadrature error control loop. The Coriolis force is detected using the analog-closed loop based on a modified PID controller, which can also be replaced with a digital control strategy based on the principle of a sigma-delta modulator (ΣΔM) for higher performance^36^. The schematic of the readout circuit is shown in Fig. 8a, in which the driving control loop, sensing control loop, and quadrature error control loop operate simultaneously. In the mode-matched gyroscopes, the frequency split greatly affects the mechanical sensitivity and bias stability of the gyroscope. For high *Q* resonators, the mechanical bandwidth is so narrow that mode matching cannot be accurately distinguished with FRA. Therefore, in this paper, the mode-matching method relies on monitoring of the Coriolis channel output in response to the quadrature electrode dither^37,38^. This method takes advantage of the fact that the quadrature leaks into the rate output only if a frequency mismatch occurs between the drive and sense mode, and the gyroscope output is proportional to the frequency mismatch. The 2 Hz sine dither signal is added to the axis-tuning voltage, and the various frequency-tuning voltages are applied on the four inner electrodes aligned with the high-frequency mode. The gyroscope output signals at several frequency-tuning voltages are shown in Fig. 8b. It can be noted that the amplitude and phase of the output are affected by the frequency-tuning voltages. With more accurate measurement, the mode-matching voltage is obtained as 30.9 V, where the output amplitude is zero, as shown in Fig. 8c.

In the readout circuit, ±12 V power voltage is used, and the signal is differentially output such that the maximum output voltage is only ±24 V. The angular inputs are set as ±1°/s, ±2°/s, ±3°/s, ±4°/s, ±5°/s, ±6°/s, ±7°/s, ±8°/s, ±9°/s, ±10°/s, ±20°/s, ±50°/s, and ±100°/s. The testing points in ±10°/s are denser than those out of ±10°/s because in our application, the range of ±10°/s is often used. As shown in Fig. 8d, the gyroscope output maintains a linear response to the rate inputs, showing a scale factor of 0.2138 V/(°/s) and a zero offset of 0.0206°/s due to the anisodamping of 3.14 × 10^4^ ppm. The bias stability performance is tested at room temperature, and the gyroscope output is recorded over 7 h. The optimized DRG shows a peak–peak noise of 4.4°/h at the sampling frequency of 1 Hz, as shown in Fig. 8e. Furthermore, the effective displacement *x*_0_ of the DRG can be derived as 2 μm by detecting the vibrating voltage in the capacitance readout circuit of the driving loop. The effective mass *m*_*eff*_ can be simulated as 2.54 mg. With the measured *Q* and resonant frequency *f*_0_, the theoretical Brownian noise ARW_Brown_ of the DRG sensor can be calculated as low as 0.001°/√h (green trace in Fig. 8f) according to the following:

7 $${\mathrm{ ARW}_{{\mathrm{Brown}}}} = \frac{1}{{2A_{\mathrm {g}}x_0}}\sqrt {\frac{{k_BT}}{{2\pi m_{eff}f_0Q}}} \times \left( {\frac{{180}}{\pi } \times 60^\circ /\sqrt h } \right)$$

Herein, *A*_g_ is the angular gain, *k*_*B*_ is the Boltzmann’s constant, and *T* is the absolute ambient temperature. Furthermore, the ARW and bias instability are tested as 0.01°/√h and 0.04°/h at room temperature without any compensation or postprocessing of data. This result shows that the ARW and bias instability of the gyroscope are dominated by the noise and drift of the readout electronics and that they must be further improved.

## Discussion

The performances of the reported MEMS DRGs and the new DRG in this paper are listed in Table 1. It can be observed that the optimized DRG presented in this work has the highest *Q* and the longest decaying time constant, and therefore the DRG has a lower ARW and a bias instability close to that of the navigation grade. However, the readout electronics severely limit the performance of the gyroscope. In our future work, the readout electronics will be improved to decrease the noise and drift.

**Table 1:** Summary of the reported DRG performances

Research institution	Sensor only		Gyroscope		Ref.	Year
	*Q* (k)	*τ* (s)	BI (°/h)	ARW (°/√h)		
UCLA	50	1.22	0.11	0.02	11	2014
UCD & SU	85	0.34	1.5	0.12	6	2014
SU	100	0.43	1.93	0.034	10	2014
UCI & SU	100	1.43	0.65	0.047	2	2014
SU	186	0.27	–	–	12	2016
Boeing	80	1.82	0.012*	0.0033*	1	2014
NUDT	358	38.5	0.08	0.012	18	2018
This paper	510	74.9	0.04	0.01	–	2018

*SU* Stanford University, *UCD* University of California, Davis, *UCB* University of California, Berkeley, *UCI* University of California, Irvine, *UCLA* University of California, Los Angeles, *NUDT* National University of Defense Technology

*Testing data under active temperature control

We present the quality factor improvement mechanism in DRGs and propose two novel methods for the quality factor improvement. With these two novel methods, the quality factor and decaying time constant of the newly designed DRG are greatly improved, giving it the potential to achieve navigation-grade performance. These quality factor improvement methods can be further applied in the optimization of other micromechanical resonators to improve their performances for high-end markets.

## Materials and methods

In this paper, COMSOL Multiphysics is chosen for simulation of *Q*_TED_ and the resonant frequency of the DRG. In the simulation, the material properties are given as follows: *E* = 170 GPa, density *ρ* = 2329 kg/m^3^, *α* = 2.6 × 10^–6^ K^–1^, *C*_*v*_ = 1.63 × 10^6^ Jm^–3 ^K^–1^, *k* = 130 Wm^–1^ K^–1^, and *T*_0_ = 293.15 K.

This disk resonator is fabricated using wafer fusion bonding and a DRIE process, as shown in Fig. 6c. A P-type 500-μm (111) single-crystal silicon wafer is used as the substrate, and an SOI wafer with a 150-μm (111) single-crystal silicon device layer is used in resonator fabrication. First, the substrate is etched via DRIE for 10 μm to form the anchors corresponding to the electrodes. Second, thermal oxidation technology is used to generate the thermal oxide layer on the substrate. Using wafer fusion bonding technology, the SOI wafer and the substrate are bonded together, and the handle wafer is moved with chemical and mechanical polishing. Finally, the aluminum wire bonding pads are patterned, and DRIE technology is applied for resonator and electrode fabrication.
